# Bee-Mediated Selection Favors Floral Sex Specialization in a Heterantherous Species: Strategies to Solve the Pollen Dilemma

**DOI:** 10.3390/plants9121685

**Published:** 2020-12-01

**Authors:** Larissa C. Oliveira, Alberto L. Teixido, Renata Trevizan, Vinícius L. G. Brito

**Affiliations:** 1 Programa de Pós-Graduação em Biologia Vegetal, Universidade Federal de Uberlândia, Campus Umuarama, Uberlândia 38405-302, MG, Brazil; 2 Departamento de Biologia Vegetal, Universidade Estadual de Campinas, Cidade Universitária, Campinas 13083-862, SP, Brazil; renatattelles@gmail.com; 3 Departamento de Botânica e Ecologia, Universidade Federal de Mato Grosso, Cuiabá E-78060-900, MG, Brazil; alberto.lopez.teixido@gmail.com; 4 Instituto de Biologia, Universidade Federal de Uberlândia, Campus Umuarama, Uberlândia 38405-302, MG, Brazil; viniciusduartina@gmail.com

**Keywords:** buzz pollination, female-biased plants, heteranthery, *Macairea radula*, male-biased plants, phenotypic gender, pollen flowers, pollen removal, stamen size, style morphology

## Abstract

Animal-pollinated plants show a broad variation in floral morphology traits and gametophyte production within populations. Thus, floral traits related to plant reproduction and sexuality are usually exposed to pollinator-mediated selection. Such selective pressures may be even stronger in heterantherous and pollen flowers, in which pollen contributes to both bee feeding and pollination, overcoming the “pollen dilemma” or the inability to perform both functions simultaneously. We describe the phenotypic gender and sexual organ morphology of flowers in two populations of *Macairea radula* (Melastomataceae), a heterantherous and buzz-pollinated species with pollen flowers. We estimated selection gradients on these traits through female and male fitness components. Both populations showed sizeable phenotypic gender variation, from strict hermaphrodites to increased femaleness or maleness. We found a continuous variation in style and stamen size, and this variation was correlated with corresponding shape values of both sexual organs. We detected bee-mediated selection towards short and long styles through seed number and towards intermediate degrees of heteranthery through pollen removal in one population, and selection towards increased maleness through pollen dispersal in both populations. Our results suggest that bee-mediated selection favors floral sex specialization and stylar dimorphism in *M. radula*, optimizing reproductive success and solving the pollen dilemma.

## 1. Introduction

Animal-pollinated hermaphroditic plants commonly show morphological variation in floral sex organs. Such morphological variations are interpreted as adaptations to avoid or, at least, optimize sexual conflicts within and between male and female functions (e.g., gamete wastage, sexual interference, self-pollination [1,2]). In fact, the huge variability of floral systems in animal-pollinated plants is generally considered as a solution to such conflicts that may happen during the interaction with pollinators [3]. Although the sexuality of animal-pollinated plants is often described by the occurrence and position of floral sex organs (stamens and carpels) in time and space, pollen and ovule production also contribute to sexual expression [4,5]. Pollen and ovule contribute disproportionately in plant reproductive success because they are not produced in the same amounts. Thus, if the plant gametophytes are taken as discrete units during the estimation of plant sexuality, hermaphroditic plants would depart from strict equisexuality [6,7], and the phenotypic gender of a plant may naturally differ from its morphological sexual expression [4,5]. Phenotypic gender may be defined as the relative proportion of all gametophytes produced by a plant as a female and male parent [4,8]. Despite the fact that phenotypic gender expression is not a novel topic in the literature, this trait has rarely been considered in studies of pollinator-mediated selection in natural populations [7,9,10,11]. Considering that floral traits of most animal-pollinated plants are under partial selection exerted by pollinators [12,13,14], it is also important to take into account their impact in plant phenotypic gender selection.

Most animal-pollinated species are pollinated by bees that, during flower visits, collect pollen as a food resource for their larvae [15,16]. Once deposited on bee scopa, pollen grains are no longer available for pollination and ovule fertilization, potentially decreasing plant male reproductive success [17,18]. Thus, there is a conflicting selection pressure on the function of pollen grains. Pollen grains may either contribute with the male gametes in the pollination process or be food for bee larvae, but they cannot perform both functions at the same time, which is broadly known as the “pollen dilemma” [18,19,20]. There is a vast number of floral adaptations that supposedly overcome this conflict, such as spiny pollen grains that hinder pollen collection [20], pollen grains that are toxic for bees [21,22], and the presence of other floral resources, such as nectar, oil, scents, and resins [23]. However, some flowers offer only pollen as a resource to motivate pollinating bees, producing a large amount of pollen grains (so-called pollen flowers [24,25]). Therefore, the phenotypic gender is expected to be an adaptive trait to solve the pollen dilemma under bee-mediated selection in pollen flowers.

In addition to increased pollen grain production, some pollen flowers also show largely specialized morphological patterns associated with sexual organs. A classic example is heteranthery (or stamen dimorphism), the occurrence of two or more groups of stamens differing in size, shape, and/or color within the same flower, an adaptation that decreases the pollen dilemma [19,26,27]. In pollen flowers, shorter stamens commonly deposit pollen on the bee ventral abdomen, an easily accessible site to active pollen collection by bees. Instead, longer stamens deliver pollen on the bee dorsal abdomen, a safer site to achieve pollination [18,19,20,28]. The evolution of heteranthery has independently occurred across 20 plant families and it is related to both pollen flower emergence and poricidal anther dehiscence [2,28]. Overall, this set of floral traits almost exclusively constrains flower visitation to some bees with the capacity of producing high-frequency vibrations to collect pollen in a specialized way in a process called buzz pollination [29,30,31,32]. Given the stamen arrangement and the pollen collection behavior of bees, style morphology and positioning are also decisive in the reproductive success of these plants [26,33,34]. In buzz-pollinated species with poricidal anther dehiscence, the grooming behavior of vibrating bees asymmetrically distributes the pollen grains in their bodies. According to the combined action of size of these stamens, the release of pollen grains, and the grooming behavior, longer styles are most suitable for pollen deposition from the bee dorsal abdomen [35,36,37]. Consequently, the size and morphology of stamens and styles entail that plants can donate and receive more or fewer pollen grains, generating a certain sexual specialization. Overall, the phenotypic gender, heteranthery degree, and style morphology may ultimately be under constant bee-mediated selective pressure in pollen flowers.

**Figure 1:**
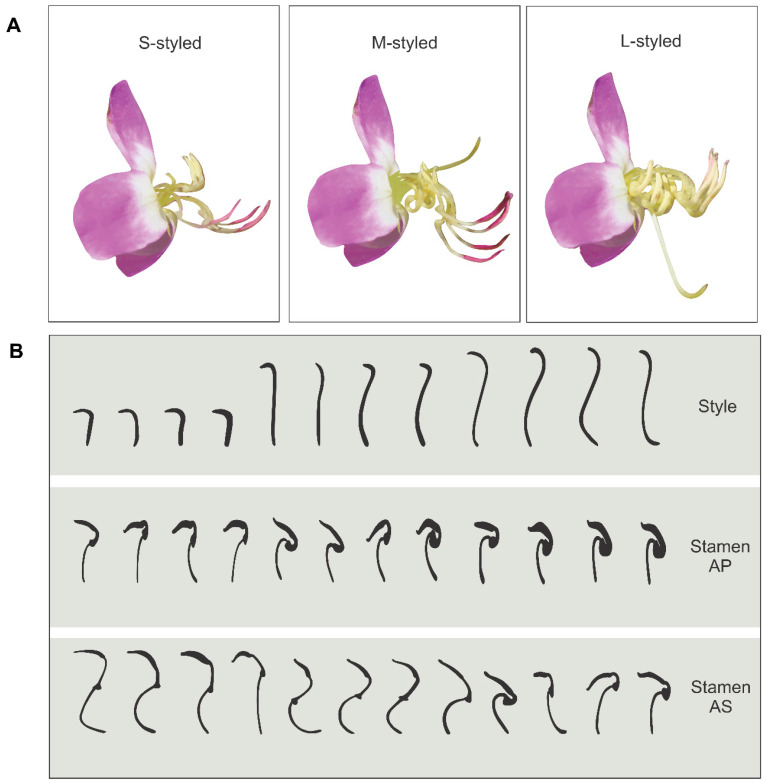
Typical floral types of *Macairea radula*. (**A**) Short-styled plants present flowers with short styles and two longer stamen sets, medium-styled plants present flowers with intermediate styles in relation to both stamen sets, and long-styled plants present flowers with styles longer than the two sets of stamens. (**B**) Scheme showing the variation in the length of sexual organs of each floral type. Each column corresponds to a flower (scale = 4 mm). AP means antepetalous stamens and AS means antesepalous stamens.

Pollen as the main floral resource, poricidal anthers, and heteranthery are buzz pollination-associated traits common in Melastomataceae [38]. Moreover, buzz pollination syndrome was likely already present in the ancestral representatives of the family [38]. Commonly, both heteranthery degree and style morphology show low levels of variation within populations of extant species of this family. However, *Macairea radula* (Bonpl.) DC. presents a high variation of such traits among individuals in polymorphic populations (Figure 1). Therefore, this is an ideal species to study vibrating bee-mediated selection on phenotypic gender, heteranthery degree, and style morphology. Studying how selection pressures act on the inter-individual variation of those floral traits can help us to understand how vibrating bees boosted flower evolution in one of the most speciose pollen-flower angiosperm clades. In this way, we aimed to understand the variation of phenotypic gender, heteranthery degree, and style morphology, in addition to bee-mediated selection on these traits in *M. radula*. Specifically, we (1) estimated and described the phenotypic gender of plants; (2) determined the morphology of sexual organs, i.e., heteranthery degree and style morphology; and (3) estimated the selection gradients exerted by vibrating bees on these traits considering both male and female reproductive success. We predict that pollinator-mediated selection pressures in *M. radula* favor sexual specialization, with traits biased to male function (e.g., increased maleness, higher heteranthery degree, and/or shorter styles) or to female function (e.g., reduced maleness, lower heteranthery degree, and/or longer styles) within populations, which would ultimately reduce the pollen dilemma.

## 2. Results

### 2.1. Phenotypic Gender

Individual plants in both populations—Delfinópolis (DEL) and Uberlândia (UDI)—varied in phenotypic gender, from male-biased to strict hermaphrodites and female-biased individuals (Figure S1). On average, however, phenotypic gender was similarly close to hermaphroditism in both DEL and UDI and did not differ between populations (Table 1; see also Table S1 for means ± SE per population). We did not find any correlation between phenotypic gender and the morphological traits of the sexual organs. (i.e., style size and heteranthery degree; Table S2).

### 2.2. Sexual Organ Morphology

We found that variation in style size is similar between populations (Table 1; see also Table S1 for means ± SE per population). However, individuals show two levels of distribution with continuous variation in style size (Figure S2A). Considering the stamens, we found that antesepalous stamens (AS) are distributed in two levels of size (i.e., short and long), whereas antepetalous stamens (AP) have only one level of size (i.e., short) (Figure S2B,C). AP stamens were significantly greater in DEL than in UDI, but AS stamens were marginally similar in both populations (Table 1; see also Table S1 for means ± SE per population). Accordingly, we did not detect any difference in heteranthery degree between populations (Table 1; see also Table S1 for means ± SE per population).

We used the first and second relative warp values, RW1 and RW2, to describe the shape of the styles and stamens. Negative RW1 values represent higher curvatures of the style insertion on the ovary, whereas positive values represent lower curvatures at the same position. Similarly, negative RW2 values are related to sigmoid shapes and positive RW2 values are related to linear shapes of styles (Figure S3). In both populations, style sizes and corresponding RW1 and RW2 values are negatively correlated (Table S3; Figure S4). For stamens, the RW1 and RW2 values describe the curve of anthers and filaments, respectively. Higher values of RW1 represent less curved anthers and lower values more curved anthers, whereas higher RW2 values describe more linear filaments and lower values more curved filaments (Figure S3). AP and AS stamen sizes were positively correlated with their RW1 and RW2 values (Table S2; Figure S4). We did not find any correlation between style size and heteranthery degree in DEL, whereas a negative correlation was detected in UDI (Table S2).

### 2.3. Phenotypic Selection

**Figure 2:**
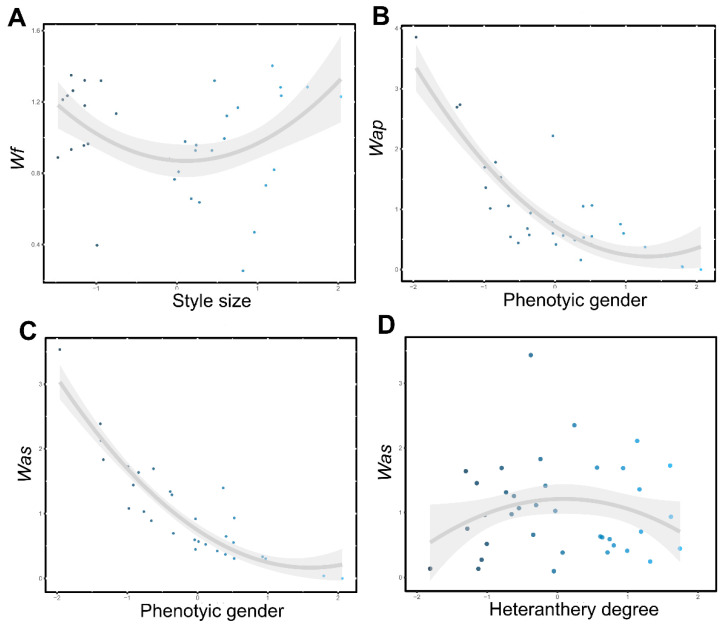
Representative surfaces of disruptive selection on style through seed number (**A**), on phenotypic gender through pollen removal of AP stamens (**B**) and AS stamens (**C**), and representative surface of stabilizing selection on heteranthery degree through pollen removal of AS stamens (**D**) in DEL population. *Wf*, *Wap* and *Was* correspond to the female and male (antepetalous stamens and antesepalous stamens, respectively) fitness components.

We found that the seed number was about 35% higher in DEL than in UDI, but pollen removal for both AP and AS stamens was similar between populations (Table 1; see also Table S1 for means ± SE per population). The estimates of phenotypic selection showed disruptive selection on style size through seed number in DEL (Table 2; Figure 2A). Considering male fitness, we detected negative linear selection on phenotypic gender through AP and AS pollen removal in both populations (Table 2), indicating higher percentage of pollen removal with increasing maleness. In DEL, we also detected that selection on phenotypic gender through AP and AS pollen removal was significantly non-linear and positive (Table 2). This pattern is associated with disruptive selection, but the relationship between phenotypic gender and male fitness showed that pollen removal rates rather tended to level off with increasing femaleness (Figure 2B,C), reinforcing the higher percentage of pollen removal in male-biased plants. Standardized non-linear selection gradients in DEL also showed a marginally significant stabilizing selection on heteranthery degree through AS pollen removal (Table 2; Figure 2D).

## 3. Discussion

Our results demonstrate that the phenotypic gender and morphology of floral sexual organs are highly variable within populations of M. radula, a heterantherous and buzz-pollinated species with pollen flowers. More interestingly, we detected that this variation translates into a significant vibrating bee-mediated selection towards flower’s sexual specialization through both seed number and pollen removal, favouring male-biased individuals that release higher amounts of pollen grains and with intermediate heteranthery degrees, and S- or L-styled individuals with high female reproductive success within populations. The factors associated to the variation in the phenotypic gender, and the morphology of floral sexual organs, such as breeding system, successional stage, pollen transfer efficiency, and reproductive success in animal-pollinated plants have been broadly considered in the literature [39,40]. However, the effects of the selection exerted by pollinators on the set of these floral traits in natural populations have so far been neglected. Ultimately, the suitable association of these traits under pollinator-mediated selective pressures seems to be an adaptive strategy that may solve the pollen dilemma.

### 3.1. Phenotypic Gender and Morphology of Sexual Organs

Although most individuals of *M. radula* express hermaphroditism in both studied populations, some individuals, as expected, depart from the strict equisexuality, expressing femaleness or maleness, that naturally differ from their sexual appearance and morphology. Phenotypic gender plays an important role in the evolution of breeding and sexual systems across plant lineages [41,42]. Thus, the variation in the expression of phenotypic gender directly reflects differences in reproductive effort between the female and male function, which in turn favors selection on these functions [4,41]. Such selection is thought to be even stronger in pollen flowers because of the twofold function related to both reproduction and feeding performed by pollen [19,20,24,25]. Our results of negative selection on phenotypic gender (i.e., selection towards maleness) through pollen removal from AP and AS stamens in both populations support this assumption and help to understand how the strategies to overcome the pollen dilemma are under significant selective pressures imposed by pollinators.

In pollen flowers, as happens in most Melastomataceae species, pollen production is a mandatory mechanism to motivate bee visitation [24]. This functional constraint may have limited the evolution of strict sexual specialization (i.e., dioecy) in the family. However, other floral resources, such as nectar, sugar corpuscles, and oils, are also found in some species within the family that modulate the interaction with alternative pollinators to buzzing bees such as birds, bats, and non-vibratory insects [43,44,45,46]. In this case, offering a floral resource different to pollen may have paved the path to the evolution of dioecy in several *Miconia* species [47,48]. In fact, in these dioecious plants, sterile anthers and nectar are the main attractant to pollinators in female flowers [48]. However, the evolutionary association between sexual systems and floral resources in Melastomataceae still needs further research to be confirmed. Nonetheless, our results demonstrate that sexual specialization can also happen in hermaphrodites by structure–function relationships in floral traits closely related to reproduction, such as the phenotypic gender and sexual organ morphology.

We verified a morphological correlation between shape and size of both styles and stamens in *M. radula*, which suggests a developmental and functional association in such organs [49,50,51]. Overall, longer stamens of *M. radula* are more sickle-shaped and less curved than shorter stamens, whereas longer styles tend to be more curved and sigmoid compared to shorter stamens. These results suggest that, ultimately, intra-individual variation of sexual organs is limited, distinguishing a narrow range of floral types within populations. These constraints in the morphology, development, and function of sex-associated floral types may, consequently, be related to individual reproductive success. In this way, we verified that both short and long styles show curves very close to the stigmatic surface, which favor pollen collection in inaccessible bee body regions [52,53]. A similar explanation can plausibly account for the relationship between stamen shape and size. Thus, more sickle-shaped and longer stamens, and less curved and shorter stamens, may deposit more pollen grains on suited and safer body regions of pollen-collecting bees, ultimately dispersing higher pollen loads.

Despite the limitations in the variation of sexual organs of *M. radula*, we found that the size and shape of styles and stamens differed between the studied populations. These interpopulation differences in the morphology of sexual organs may indicate two nonexclusive processes: local adaptation and/or phenotypic plasticity, both potentially associated with the differences in environmental conditions between populations. Because the morphological match between bees and stamens is crucial to increase outcrossing in pollen flowers [54], it is not implausible to think that any difference in bee diversity between localities would readily lead to differences in sizes and shapes of the sexual organs. Likewise, populations may differ in the availability of essential environmental resources, such as water and nutrients, which also influence the phenotypic expression of pollination-related floral traits [55]. However, more data are necessary to make solid conclusions about the morphological differences in floral sexual organs between populations of *M. radula*.

### 3.2. Phenotypic Selection

As predicted, we detected that selection favored male phenotypic genders through pollen release from both AP and AS stamens. This result reinforces the assumption that floral sex specialization in *M. radula* is selected by differential bee-mediated pollen removal among individuals within populations. Because we used the mean percentage of pollen removed per plant as a proxy for an indirect estimate of male reproductive success, our results also demonstrate that, regardless of heteranthery degree, vibrating bees collect more pollen grains in male-biased individuals of *M. radula* from shorter (AP) stamens that would contribute to food for larvae. Simultaneously, and as an inherent process, the same bees would also remove disproportionally higher amounts of pollen intended for pollination process (i.e., from longer, AS stamens) from such flowers than in plants with increased femaleness.

However, contrary to our expectations, we did not detect a positive linear selection on heteranthery degree through pollen removal. Rather, we detected a marginal stabilizing selection on this trait through pollen removal from AS stamens in DEL, which favors individuals with intermediate values of heteranthery degree in this population. In *M. radula*, low heteranthery degree corresponds to reduced sizes of both stamen types, facilitating pollen adherence to the bee ventral abdomen and its subsequent use as a food resource, whereas in flowers with higher heteranthery degrees, a marked dimorphism in stamen size would potentially promote male fitness by increasing pollen attachment to the bee dorsal abdomen and ensuring pollen dispersal [37]. Our findings for heteranthery degree, particularly for stabilizing selection despite the marginal significance, are difficult to explain. The most plausible explanation for this pattern is simply that male-biased individuals of *M. radula*, which show high rates of pollen removal and subsequent bee-mediated positive selection, tend to show intermediate heteranthery degrees in DEL. We are aware that some methodological limitations in the estimates of phenotypic selection may account for our results. In this regard, pollen removal does not strictly represent seed siring and molecular analyses with genetic markers are required to record reliable estimates of male fitness [56]. Therefore, interpretations for selection through pollen removal should be made with caution.

We also found disruptive selection on style size through seed number in DEL. In this population, flowers with short and long styles set relatively more seeds than medium-styled flowers. Extreme values for style size may have been selected by their higher accuracy in pollen receipt because they better match both the dorsal and ventral parts of the bee abdomen than medium-sized styles [37,57]. Accordingly, the high accuracy in style size in DEL may explain the higher seed production in this population in relation to UDI. Diversifying selection on style size may ultimately drive the evolution of stylar dimorphism, a floral system in which flowers with short and long styles are more common than medium styles within a given population [58]. However, stylar dimorphism is different from heterostyly mostly because reciprocal herkogamy is not guaranteed in the former case [58,59,60,61]. Stylar dimorphism associated with a relaxed heteranthery, wherein stamen length is non-reciprocal to style length, could be a more plausible explanation to the high variation of sexual organs in *M. radula* than the heterostyly previously interpreted for this species [37].

In conclusion, *M. radula* is a typical pollen flower species with a highly specialized pollination system involving vibrating pollen-collecting bees. The offering of pollen as the main resource to motivate bee visits generates trade-offs between feeding and pollination functions [18,19]. In this scenario, strategies that increase the morphological match with bees and structure–function relationships in floral traits closely related to this process, such as the phenotypic gender, heteranthery degree, and style morphology, should be favored once they potentially diminish these conflicting demands. Interestingly, our results demonstrate that these floral traits, at least partially, are selected by buzzing bees through male and female fitness components in *M. radula*. Plants with increased maleness and individuals with short or long styles, biased to female function, are selected, possibly to (1) optimize reproductive success by increasing effective pollen dispersal and pollen receipt, respectively, and (2) to solve the pollen dilemma by partitioning the pollen load for both feeding and pollination demands. Therefore, bee-mediated selection favors floral sex specialization and stylar dimorphism in *M. radula*, a heterantherous and buzz-pollinating species with pollen flowers inhabiting a Neotropical savanna ecosystem.

## 4. Material and Methods

### 4.1. Study Sites and Species

Fieldwork was conducted between August 2017 and November 2018 during the flowering peak of two *M. radula* populations approximately 340 km apart in Minas Gerais State, Brazil: DEL—Fazenda Águas de Santo Antônio (20°25′ S 46°40′ W; 844 m a.s.l; Delfinópolis municipality) and UDI—Fazenda Ourinhos (19°03′ S 48°21′ W; 801 m a.s.l; Uberlândia municipality). Both populations comprise many herbs and shrubs from families Poaceae, Cyperaceae, Asteraceae, and Melastomataceae. The climate is Cwa type in DEL with a mean temperature of 18 °C and about 1250 mm of annual precipitation. In UDI, the climate is Aw type, with annual mean temperature of 22 °C and annual precipitation of 1500 mm [62].

*Macairea radula* is a common shrub plant found in the veredas of the Cerrado (Brazilian savanna), which are characterized by palm swamps with a dense herb-subshrub dominant layer [63]. This species presents bisexual flowers that are organized in inflorescences and offer pollen as the main floral resource. Anthesis starts around 7:00 and flowers remain reproductive for two days. Each flower presents the ovary divided in four locules and contains 221.5 ± 43.7 ovules (mean ± SD), and the anthers produce 310,439.8 ± 247,992.9 pollen grains. The flower pollen:ovule ratio is, on average, about 1401.1 and each fruit bears 154.7 ± 55.9 seeds (LCO personal observation). Flowers have one sigmoid style and two sets of four stamens arranged in a shorter set (AP stamens), and a longer set (AS stamens) with sickled poricidal anthers (Figure 1). Length and shape of both styles and stamens largely vary among plants within populations [64]. In general, it is possible to distinguish individuals with one of the following three different types of flowers: (1) short styles and two longer stamen sets (S-styled plants); (2) intermediate styles in relation to the stamen sets (M-styled plants); and (3) plants with styles longer than the two sets of stamens (L-styled plants) (Figure 1A) [37]. This variation in sexual organ morphology has been previously interpreted as a case of heterostyly, a floral polymorphism characterized by reciprocal positioning of stigma and anther heights between different morph types [65]. In addition to the sexual organ arrangement in two (distyly) or three (tristyly) levels, plants with this mechanism are commonly related with self- and intramorph-incompatibility systems associated with reciprocal herkogamy [66]. Although three floral types can be visually discriminated in the *M. radula* populations, the variation in the length of sexual organs is not arranged in reciprocal levels, and plants are self-compatible and present a low degree of reciprocal herkogamy (Figure 1B) [37]. Overall, these reproductive characteristics refute the hypothesis that populations are heterostylic (tristylic) and indicate that the heritability of sexual organs in *M. radula* occurs by multiple-locus segregation [37]. Therefore, analysis of pollinator-mediated selection on continuous traits can be employed in this system.

Bees of the genera *Andrena, Bombus* and *Centris* are commonly seen vibrating *M. radula* flowers. These species are considered effective pollinators given the high morphological matching between their bodies and the floral sex organs. Considering the high variability in sexual organ morphology, pollen transfer may occur in both the bee ventral and dorsal abdomen [37]. Vibrating bees perform a stereotypical grooming behavior that generates an asymmetric distribution of pollen load on their body. Grooming happens mostly in the bee ventral abdomen, whereas the bee dorsal abdomen remains as a safer site for pollination [52,53]. Nevertheless, it is possible that short styles also receive the remaining pollen grains left on the bee ventral abdomen after grooming behavior. Therefore, L-styled plants should act more as pollen receptors (female-biased individuals) because their stigmas contact with such safe sites and each stamen set deposits pollen grains in the bee ventral abdomen. Conversely, S- and M-styled flowers should act more as pollen donors (male-biased individuals) because one stamen set will be directly in contact with the bee dorsal safe site while their stigmas contact the ventral region.

### 4.2. Phenotypic Gender

We randomly chose 39 and 37 *M. radula* plants in DEL and UDI populations, respectively, to describe their phenotypic gender. We collected three buds in pre-anthesis and three 2-day flowers in which the probability of bee visits is greater given the longer exposure time of each individual flower. These flowers can be characterized by changes in the color of their sexual organs due to floral senescence. Normally, 2-day flowers of *M. radula* are not visited by vibrating bees, as occurs in other species of Melastomataceae that show changes in floral color [67,68]. For each individual, we estimated the quantity and viability of pollen grains of all AP and AS stamens of three flowers for each stage, i.e., buds and 2-day flowers (n = 76 individuals × 2 stages × 3 flowers × 4 AS stamens × 4 AP stamens = 7296 stamens). The number of pollen grains was estimated using a hemocytometer and 10 µL of the total of a solution with 200 µL of acetic carmim, wherein pollen grains of each anther were homogenized [37]. Pollen viability was taken from the staining of the pollen cytoplasm in a 1.2% solution [69]. The same procedure was used to quantify the total number of ovules.

We used the number of viable pollen grains (male reproductive effort) and ovules (female reproductive effort) to describe the phenotypic gender (G) of each individual. Phenotypic gender measures were estimated using Lloyd’s quantitative femaleness method [4] (1):

(1) $$G=\frac{di}{di+\;li.\;\frac{\sum_{i}di}{\sum_{i}di}\;}\;$$

where, *d_i_* corresponds to the number of ovules and *l_i_* to the number of pollen grains of the individual *i*. This measure uses a standardized scale ranging from 0 to 1, with the average of all individuals weighted by their total fitness always equal to 0.5 [4]. Thus, G values close to 1 represent a phenotypic gender with higher femaleness (i.e., female-biased plants), whereas values close to 0 represent a phenotypic gender with higher maleness (i.e., male-biased plants). Values close to 0.5 represent cases of perfect hermaphroditism in the population.

### 4.3. Sexual Organ Morphology

We used the same individuals to describe the size and shape of their sexual organs using geometric morphometric analysis [49,70]. In addition to size measurements, conventionally used in many studies, we chose to insert measurements of shape because they also have the potential to influence plant reproductive success. In each plant, we collected three fully recently open flowers in different inflorescences, totaling 117 and 111 flowers analyzed in DEL and UDI, respectively. Style and stamens of each group of three flowers (representing one individual plant) were separated and prepared on a glass plate with graph paper (Figure S5). Each of these sets was then photographed with a digital camera (Canon EOS30D) on a regular tripod coupled with an interchangeable lens (EF-S 18-135mm f/3.5-5.6 IS STM). During photograph sections, the lens was carefully positioned in parallel to the glass plate and its focal distance was adjusted to 50 mm to minimize distortions of sexual organs that could happen due to the photographic equipment.

Photographs were organized in tpsUtil software [71] and landmarks and semi-landmarks were taken in tpsDig software [71]. Marks were defined by the homology of the coordinates in bi-dimensional space, representing the common morphology found in different plants. We defined 10 anatomical marks divided in each reproductive structure. Marks 1 to 4 refer to the pore of the anthers, the insertion of anthers in the stamen connective, the insertion of the filament in the connective, and the insertion of the filament in the flower hypanthium of the AS stamen, respectively (Figure S5). Following the same basis, marks 5 to 8 refer to the same floral structures on the AP stamen (Figure S5). Marks 9 and 10 refer to the stigmatic surface and the insertion of the style on the ovary apex, respectively. Semi-landmarks were defined as equally spaced coordinates among the marks along the contour of stamens (9 semi-landmarks) and style (8 semi-landmarks) (Figure S5).

Shape variables were generated in the geomorf package through a Generalized Procrustes Analysis (GPA) [70] in R software [72]. In GPA analysis, the shape of each structure can be represented by a point in space in a non-Euclidean way. Relative warp values (RW) were obtained from a Principal Component Analysis (PCA) in the variance–covariance matrix resulting from GPA and used as measures of variation in the shape of each structure in each individual. The centroid size (i.e., the square root of the sum of the squared distances of the anatomical landmark *i* in relation to the centroid of the points) was used as a dimensionless measure of the size of these structures [49,50,70]. Because size and shape were correlated with each other for both styles and stamens in both populations (Table S2), we used style, AP stamen size, and AS stamen size as representative floral morphological traits of *M. radula*. From the difference in the size of the centroid of AP and AS stamens, we subsequently estimated the degree of heteranthery.

### 4.4. Phenotypic Selection

To estimate female reproductive success, we picked three ripe fruits before seed dispersal per plant in each population. We subsequently quantified the number of seeds for each fruit and individual plant and then averaged them (± SD) to obtain a mean number of seeds per fruit for each plant (i.e., seed number, our estimate of female reproductive success). Seed number is a good proxy to assess the magnitude of pollen limitation and resulting female reproductive success [73]. Given that there is a negative relationship between the number of removed pollen grains and pollen limitation in flowers with poricidal anthers [74], we used the mean difference between the number of viable pollen grains of floral buds and the number of viable pollen grains that remained in 2-day old flowers as a proxy of the mean percentage of pollen removed per plant and, ultimately, as an indirect estimate of male reproductive success. The amount of removed pollen represents a useful indirect measure to disentangle the mechanisms, strength, and direction of phenotypic selection through male function [75,76,77]. Male reproductive success was estimated for both AP and AS stamens because in heterantherous buzz-pollinated flowers of Melastomataceae both stamen sets are potentially under different selective pressures, i.e., pollen grains contributing to either food for bee larvae or the pollination process.

### 4.5. Statistical Analysis

For each population, we tested the correlation between style, and AP and AS stamen size with their corresponding shape (relative warp values, RW1 and RW2) using Spearman’s rank-order correlation. A similar procedure was used to test for correlations between phenotypic gender, heteranthery degree, and style size for each population. The assumptions of normality and homogeneity of variance of each variable were tested using the Shapiro–Wilk and Levene tests, respectively.

To test for differences in the size of styles and stamens (both AP and AS), phenotypic gender, and heteranthery degree (dependent variables) between populations (fixed factor), we fitted five models (one for each dependent variable) by means of Generalized Linear Mixed Models (GLMMs), including plant nested within populations (random factor). For all variables we assumed a quasi-Poisson error distribution because of overdispersion of data with a log link function [78]. The models were analyzed using the restricted maximum likelihood (REML). To test the effect of the population on seed number and pollen removal (in both AP and AS stamens), we fitted three additional GLMMs including plants nested within populations as random factor. We assumed a normal error distribution with an identity link function for seed number and a quasi-Poisson error distribution for AP and AS pollen removal to avoid overdispersion. All GLMMs were conducted using the MASS package [79] in R software [72].

To estimate phenotypic selection on phenotypic gender, heteranthery degree, and style morphology through male and female components of reproductive success per population, we assessed standardized selection gradients (β) using linear multiple regression analyses with relative pollen removal of AP stamens, relative pollen removal of AS stamens, and relative seed number (all calculated as individual fitness/population mean fitness, *w*) as the response variable, separately, and standardized values of phenotypic gender, heteranthery degree and style morphology (with a mean of 0 and a variance of 1) as explanatory variables [80]. Correlational selection between floral traits (phenotypic gender × heteranthery degree, phenotypic gender × style morphology, and heteranthery degree × style morphology) were also estimated. Additionally, we calculated non-linear selection gradients (γ) to estimate stabilizing/disruptive selection by obtaining quadratic deviations from the mean for both single and quadratic terms of phenotypic gender, heteranthery degree, and style morphology [80]. Quadratic regression coefficients were doubled to properly estimate stabilizing/disruptive selection gradients [81]. All models were performed using the R software [72].

## Figures and Tables

**Table 1:** Summary table of the Generalized Linear Mixed Models (GLMMs) used for the studied floral traits between populations and plants (random factor nested within populations). Phenotypic gender, style size, AP and AS stamen size, heteranthery degree, and AP and AS pollen removal were modeled as a quasi-Poisson error distribution. Seed number was modeled as a Gaussian error distribution. AP means antepetalous and AS means antesepalous. Sample size was taken from three flowers of 35 individuals in the Uberlândia (UDI) population (N = 105) and 37 individuals in the Delfinópolis (DEL) population (N = 117). Significant *p*-values are bolded.

Trait	σ^2^ ± SE	Df	F	*p*
Phenotypic gender				
Population		1	3.321	0.073
Plant (Population)	0.158 ± 0.189			
Style size				
Population		1	0.314	0.577
Plant (Population)	0.277 ± 0.072			
AP stamen size				
Population		1	5.798	**0.018**
Plant (Population)	0.173 ± 0.057			
AS stamen size				
Population		1	3.246	0.076
Plant (Population)	0.199 ± 0.075			
Heteranthery degree				
Population		1	0.109	0.741
Plant (Population)	0.212 ± 0.067			
Seed number				
Population		1	17.400	**<0.001**
Plant (Population)	29.267 ± 37.800			
AP pollen removal				
Population		1	0.081	0.777
Plant (Population)	0.383 ± 264.189			
AS pollen removal				
Population		1	0.140	0.709
Plant (Population)	0.336 ± 229.725			

**Table 2:** Standardized selection gradients (directional, β*i*; quadratic, γ*_ij_* and correlational, γ*_ij_*) for floral traits and their correlation in *Macairea radula* through the female (*w*f) and male (*w*ap—antepetalous stamens—and *w*as—antesepalous stamens–) fitness components in the two studied populations.

					Trait j	
					Heteranthery Degree	Phenotypic Gender
		Trait i	β*i* ± SE	Ƴ*i* ± SE	Ƴ*ij* ± SE	Ƴ*ij* ± SE
DEL		Phenotypic gender	0.005 ± 0.072	0.079 ± 0.093	−0.073 ± 0.067	‒
	*W*f	Style size	0.115 ± 0.124	0.553 ± 0.266 ^a^	0.198 ± 0.129	−0.009 ± 0.077
		Heteranthery degree	0.050 ± 0.078	−0.059 ± 0.165	‒	‒
	*W*ap	Phenotypic gender	−0.718 ± 0.133 ***	0.482 ± 0.179 *	0.128 ± 0.126	‒
		Style size	−0.073 ± 0.215	0.037 ± 0.504	0.034 ± 0.223	0.151 ± 0.151
		Heteranthery degree	−0.063 ± 0.139	0.310 ± 0.291	‒	‒
	*W*as	Phenotypic gender	−0.635 ± 0.093 ***	0.464 ± 0.123 **	−0.086 ± 0.087	‒
		Style size	0.200 ± 0.151	0.568 ± 0.348	0.206 ± 0.156	0.012 ± 0.103
		Heteranthery degree	0.102 ± 0.095	−0.354 ± 0.199 ^b^	‒	‒
UDI		Phenotypic gender	0.028 ± 0.070	0.169 ± 0.135	−0.101 ± 0.075	‒
	*W*f	Style size	0.057 ± 0.126	0.055 ± 0.146	0.119 ± 0.183	0.065 ± 0.063
		Heteranthery degree	0.045 ± 0.090	0.138 ± 0.190	‒	‒
	*W*ap	Phenotypic gender	−0.489 ± 0.118 ***	0.048 ± 0.210	0.029 ± 0.128	‒
		Style	0.114 ± 0.197	0.152 ± 0.233	0.107 ± 0.281	0.031 ± 0.098
		Heteranthery degree	0.049 ± 0.152	0.005 ± 0.305	‒	‒
	*W*as	Phenotypic gender	−0.367 ± 0.078 ***	−0.114 ± 0.152	0.032 ± 0.084	‒
		Style size	−0.037 ± 0.142	0.038 ± 0.164	−0.154 ± 0.205	0.054 ± 0.071
		Heteranthery degree	0.080 ± 0.102	−0.159 ± 0.213	‒	‒

Significant gradients are represented by: ^a^ *p* = 0.05; ^b^ *p* = 0.08; * *p* < 0.05; ** *p* < 0.01; *** *p* < 0.001.

## Supplementary Materials

The following are available online at https://www.mdpi.com/2223-7747/9/12/1685/s1, Table S1. Mean ± SE of the measurements recorded for each studied trait of flowers of *Macairea radula* in the two populations of study (DEL and UDI). Size was based on the centroid size as a dimensionless measure. AP means antepetalous stamens and AS means antesepalous stamens. Different letters show significant differences between populations (see Table 1). Table S2. Spearman’s correlation between floral traits used to estimate phenotypic selection in the two studied populations (DEL and UDI). Sample size was taken from 35 individuals in UDI and 37 individuals in DEL. * *p* = 0.0219. Table S3. Spearman’s correlation between size and its corresponding values of shape (relative warp values—RW) of styles and stamens of *Macairea radula* flowers of the two studied populations (DEL and UDI). AP means antepetalous stamens and AS means antesepalous stamens. Sample size was taken from 35 individuals in UDI and 37 individuals in DEL. * *p* < 0.05; ** *p* < 0.001. Figure S1. Phenotypic gender distributions of the flowers of *Macairea radula* occurring in the studied populations (DEL and UDI). Figure S2. Distributions of the centroid size for styles, antepetalous and antesepalous stamens of the flowers of *Macairea radula* occurring in the studied populations (DEL and UDI). Figure S3. First and second relative wraps (RW1 and RW2) distribution representing the shape of styles, and antepetalous and antesepalous stamens of the flowers of *Macairea radula*. Schemes in black represent the maximum and minimum values. Figure S4. Correlation between the first and second relative wraps (RW1 and RW2) and the size of styles, and antepetalous and antesepalous stamens of the flowers of *Macairea radula* occurring in the studied populations (DEL and UDI). Figure S5. Anatomical marks (red points) and semi-landmarks (black points) considered for the description of shape and size of each reproductive structure of the flowers of *Macairea radula* occurring in the studied populations (DEL and UDI).
