# Supplementary material for: Bee-Mediated Selection Favors Floral Sex Specialization in a Heterantherous Species: Strategies to Solve the Pollen Dilemma
Source: Plants (Basel). 2020 Dec 1;9(12):1685. doi: 10.3390/plants9121685 (PMC7760250; doi:10.3390/plants9121685)
Supplement: Supplementary file 1 [file plants-09-01685-s001.zip › plants-1002239-supplementary/plants-1002239 - Tables.pdf]

Table S1. Mean ± SE of the measurements recorded for each studied trait of flowers of *Macairea radula* in the two populations of study (DEL and UDI). Size was based on the centroid size as a dimensionless measure. AP means antepetalous stamens and AS means antesepalous stamens. Different letters show significant differences between populations (see Table 1).

Trait	DEL	UDI
Phenotypic gender	0.634 ± 0.149[a]	0.557 ± 0.132[a]
Style size	0.929 ± 0.245[a]	0.965 ± 0.214[a]
AP stamen size	0.731 ± 0.146[a]	0.623 ±0.094[b]
AS stamen size	1.349 ± 0.248[a]	1.186 ± 0.209[a]
Style RW1	-0.023 ± 0.071[a]	0.024 ± 0.063[b]
Style RW2	-0.0005 ± 0.037[a]	0.0002 ± 0.036[a]
AP stamen RW1	-0.072 ± 0.107[a]	-0.176 ± 0.114[b]
AP stamen RW2	-0.067 ± 0.070[a]	-0.017 ± 0.091[b]
AS stamen RW1	0.166 ± 0.079[a]	0.077 ± 0.141[b]
AS stamen RW2	0.028 ± 0.051[a]	0.056 ± 0.064[a]
Heteranthery degree	0.293 ± 0.066[a]	0.301 ± 0.0710[a]
Seed number	177.9 ± 44.2[a]	131.5 ± 34.9[b]
AP pollen removal	176,838 ± 124,142[a]	168,215 ± 93,909[a]
AS pollen removal	140,843 ± 85,845[a]	124,670 ± 72,629[a]

Table S2. Spearman's correlation between floral traits used to estimate phenotypic selection in the two studied populations (DEL and UDI). Sample size was taken from 35 individuals in UDI and 37 individuals in DEL. *p= 0.0219.

Correlation	DEL	UDI
Style size x Heteranthery degree	0.077	-0.223*
Style size x Phenotypic gender	0.004	-0.094
Heteranthery degree x Phenotypic gender	0.125	0.054

Table S3. Spearman's correlation between size and its corresponding values of shape (relative warp values – RW) of styles and stamens of *Macairea radula* flowers of the two studied populations (DEL and UDI). AP means antepetalous stamens and AS means antesepalous stamens. Sample size was taken from 35 individuals in UDI and 37 individuals in DEL. *p < 0.05; ** p < 0.001.

Population	Variable	RW1	RW2
DEL	Style size	-0.628**	-0.456**
	AP stamen size	0.588**	0.540**
	AS stamen size	0.586**	0.441**
UDI	Style size	-0.449**	-0.398**
	AP stamen size	0.374**	0.356**
	AS stamen size	0.682**	0.194*
