# Supplementary figures and images for: Bee-Mediated Selection Favors Floral Sex Specialization in a Heterantherous Species: Strategies to Solve the Pollen Dilemma
Source: Plants (Basel). 2020 Dec 1;9(12):1685. doi: 10.3390/plants9121685 (PMC7760250; doi:10.3390/plants9121685)

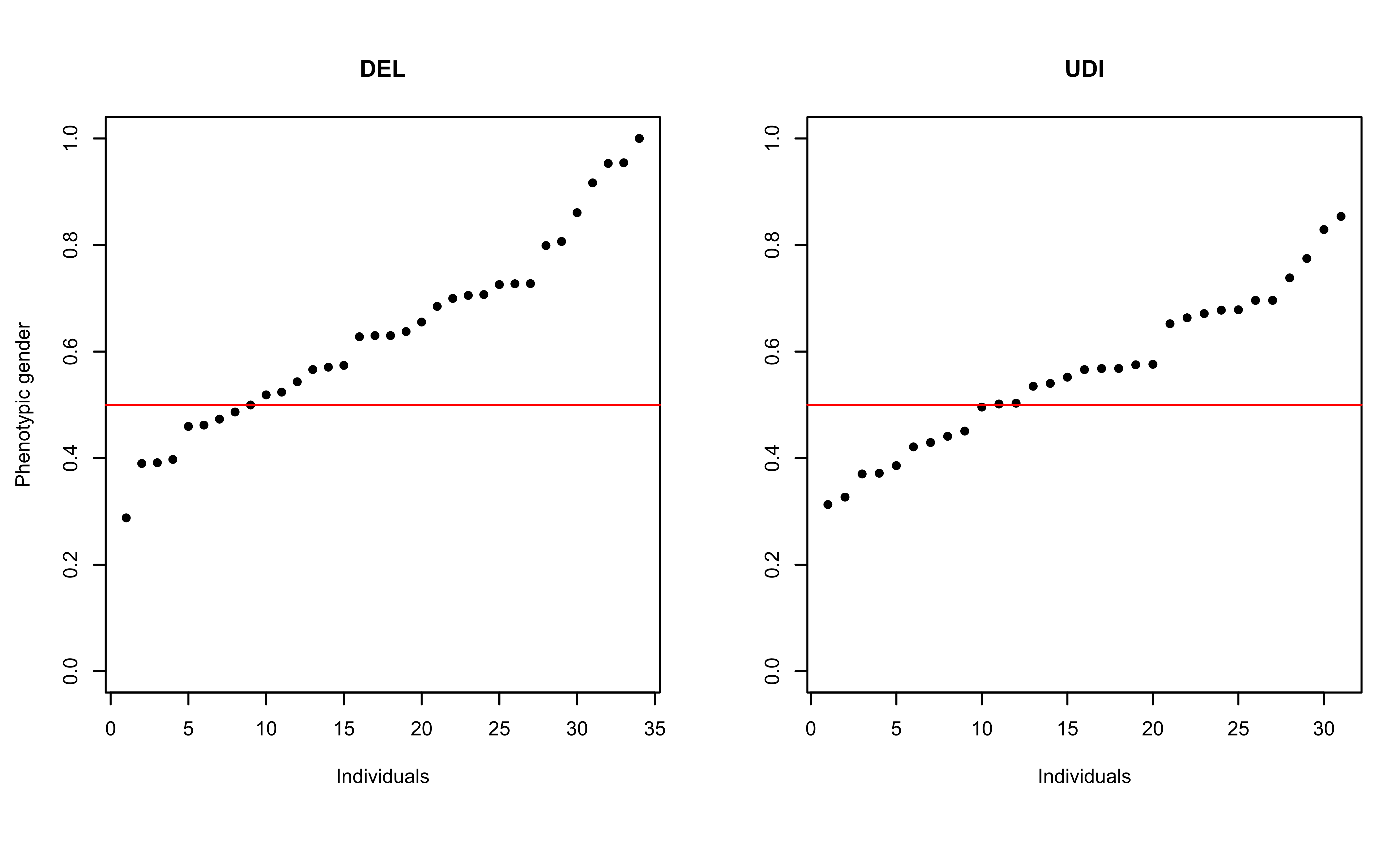

Supplement: Supplementary file 1 [file plants-09-01685-s001.zip › plants-1002239-supplementary/Figure S1.tif]

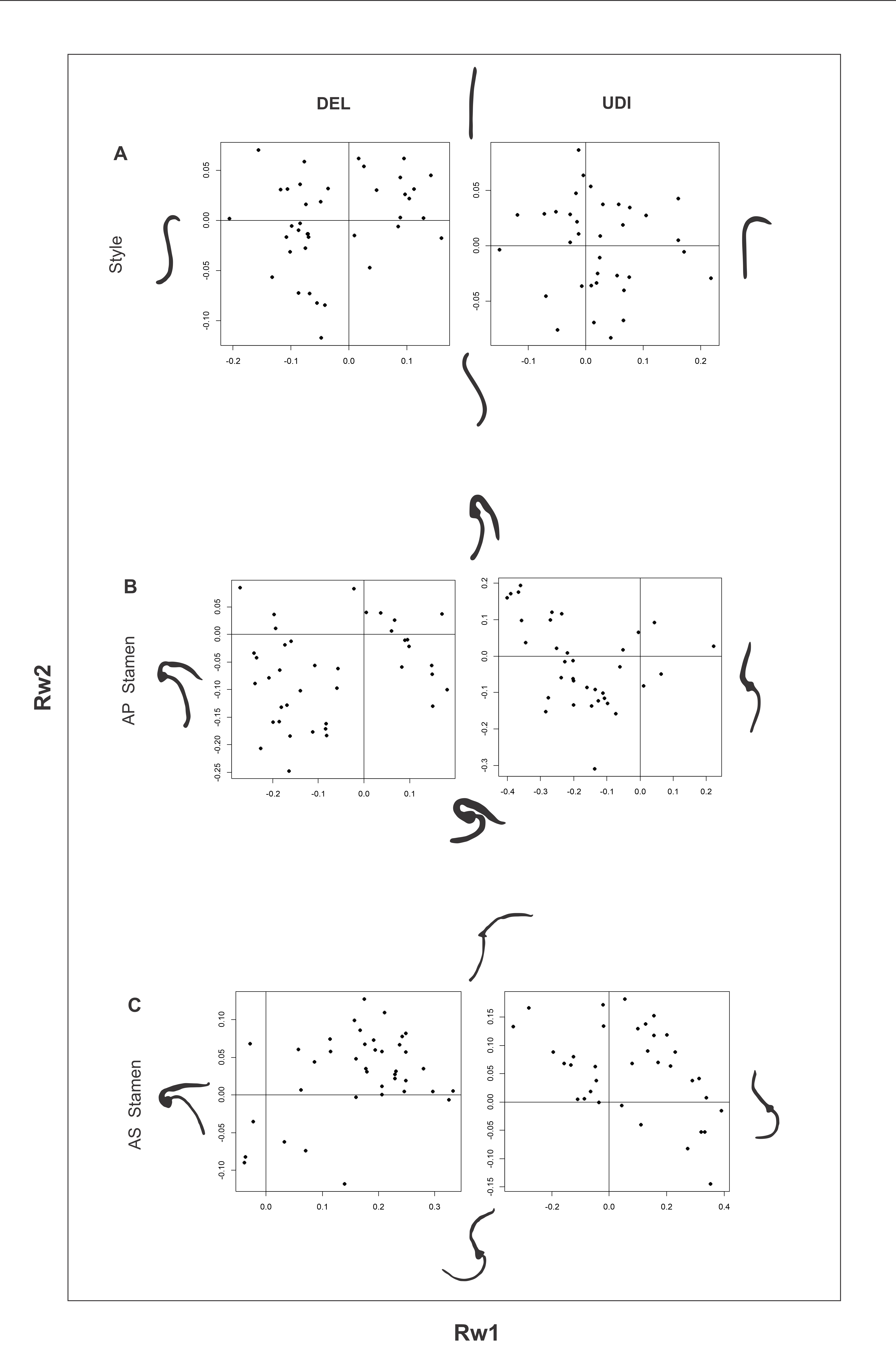

Supplement: Supplementary file 1 [file plants-09-01685-s001.zip › plants-1002239-supplementary/Figure S3.tif]

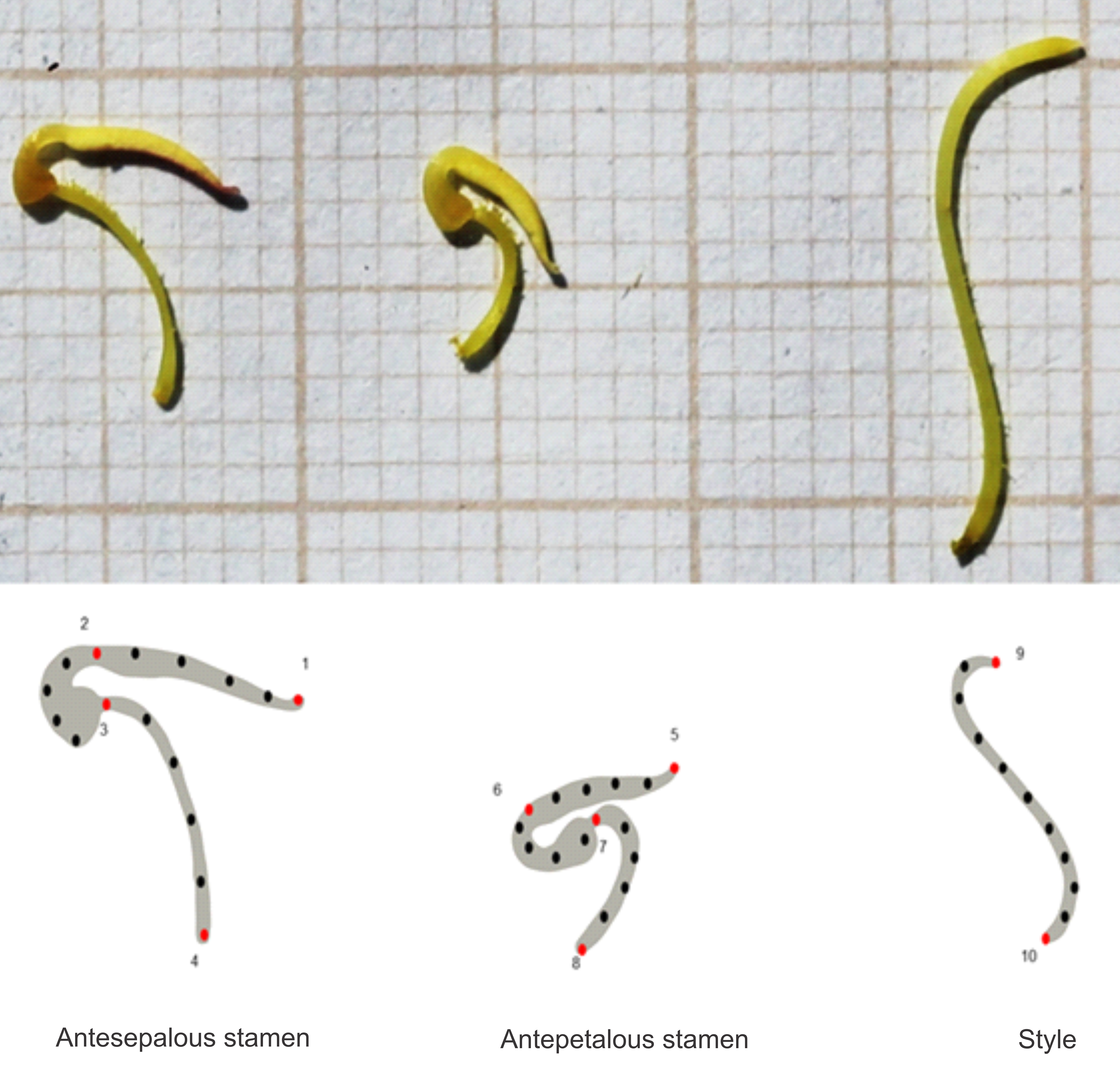

Supplement: Supplementary file 1 [file plants-09-01685-s001.zip › plants-1002239-supplementary/Figure S5.tif]
